# Synergistic Tribofilm Growth in Ethylene Glycol: A Dual-Additive Approach for Superior Lubrication

**DOI:** 10.3390/ma19030493

**Published:** 2026-01-26

**Authors:** Xiangli Wen, Peng Gong, Ningyi Yuan, Yu Tian, Lvzhou Li, Jianning Ding

**Affiliations:** 1 Institute of Technology for Carbon Neutralization, Interdisciplinary Research Center for Advanced Energy, Yangzhou University, Yangzhou 225127, Chinadingjn@yzu.edu.cn (J.D.); 2 College of Mechanical and Vehicle Engineering, Chongqing University, Chongqing 401331, China; 3 Jiangsu Collaborative Innovation Center for Photovoltaic Science and Engineering, Changzhou University, Changzhou 213164, China; 4 State Key Laboratory of Tribology, Department of Mechanical Engineering, Tsinghua University, Beijing 100084, China

**Keywords:** ethylene glycol hydraulic fluid, tribofilm, synergistic effect, extreme pressure, wear resistance

## Abstract

This study provides an original insight into the synergistic mechanism through which TM-104 and Vanlube 672 facilitate the in situ formation of a nanoscale bilayer tribofilm in ethylene glycol-based hydraulic fluid. By optimizing the additive formulation to 0.5 wt.% TM-104 and 2.0 wt.% Vanlube 672, a structurally graded tribofilm was autonomously assembled at the friction interface, comprising a 6 nm-thick P_x_O_y_-rich inner layer and a 140 nm-thick amorphous carbon outer layer. This engineered interlayer delivers exceptional tribological enhancements, with a 31% improvement in lubricity, a 71% increase in wear resistance, and a remarkable 577% enhancement in extreme-pressure load capacity. The first discovery was that there were differences in the mechanisms between these two layers: the inner P_x_O_y_ layer establishes strong chemisorption bonds with the substrate, while the outer carbon layer facilitates energy dissipation through shear-induced graphitization. These findings establish a new paradigm for designing multi-functional lubricant additives and provide a scientific basis for developing high-performance fire-resistant hydraulic fluids operable under extreme conditions.

## 1. Introduction

Water-based fire-retardant hydraulic fluid (HFC) is an ideal fire-retardant hydraulic medium with good flame retardancy, safety, stability, and environmental friendliness [1,2]. HFC is the most important type of water-based hydraulic fluid [3], with notable advantages that include low environmental impact, efficient heat transfer capacity, compatibility with rubber, shear stability, stability in storage, and low cost [4,5]. HFC can be used in hydraulic systems such as marine equipment [6], aviation starters [7], and mining equipment [8], as well as in daily skin care products [9] and antifreeze [10]. As the main component of HFC, ethylene glycol (EG) aqueous solution exhibits excellent flame retardancy, low-temperature fluidity, and thermal conductivity, and ensures basic lubrication under low loads [11,12]. However, its low viscosity and easy oxidation hinder the formation of a stable lubricating film on the surface interface of hydraulic system equipment. HFC is prone to bubble erosion and friction corrosion under service conditions, which cause system vibration and wear failure and can significantly affect the safety and service life of equipment [13,14,15]. Electrolytic corrosion can easily occur when impurities or some additives are present in seawater [16,17]. For example, corrosion can be exacerbated in 316L stainless steel under higher temperatures, penetration by impurity ions (Na^+^, Mg^2+^, Cl^−^, etc.), or increased concentrations of EG [17].

Many studies on modifications to additives have been conducted on the main components of aqueous HFC and EG solutions using ionic liquids [18,19], nanoadditives [20,21], and organic additives [22,23]. Water-soluble organic Mo- and P-containing additives have also been widely used in water-based lubricants and alcohol substances to effectively reduce friction, wear, and corrosion in boundary lubrication systems [24,25]. Water-soluble Mo effectively reduces the friction coefficient of pentaerythritol, improves its antiwear performance, and broadens the range of temperatures in which it can be applied [26]. The frictional properties of aqueous solutions can be improved by transforming the composition of the friction reaction film generated by organic Mo on the surface of steel (FeS → MoS_2_) [27]. Polyethylene glycol modification enhances the dispersibility of SiO_2_ particles, enriches organic Mo in the friction contact zone to form a stable adsorption film, and improves the high-temperature lubrication performance of water-based drilling fluids by more than 70% [28]. Additionally, the synthesis of P-containing quantum dots via high-energy ball milling achieved macroscopic superlubricity at 336 MPa through rolling effects and lower shear forces, although this performance was limited to a lower range of loads [29]. The generation of a P_x_O_y_ reaction film with a thickness of approximately 90 nm by the frictional reaction of the P-quantum dots can lead to excellent tribological properties, which are nonetheless limited to lower operating speeds [30]. A comparison of the tribological properties of P-containing additives in EG solution showed that longer alkyl chains enabled easier self-assembly and the formation of a physical adsorption film on the friction surface while promoting the formation of a reaction film. Thus, they maintained excellent tribological properties at high loads and frequencies [31]. However, a single additive only enhances the antifriction and antiwear effects of an EG aqueous solution within a limited range and is largely ineffective under extreme working conditions or in marine environments [32]. Single additives also involve some notable problems with their solubility, dispersibility, corrosiveness, durability, and resistance to high humidity [33,34,35]. The synergistic effect of compound additives can form a reaction film in a friction contact area, which further affects the lubrication, wear, and corrosion properties of lubricating media [36,37]. Wang et al. verified that the interlayer shear effect and the adsorption of Mo-P composite additives on the friction surfaces of the friction pair are the key factors for reducing the coefficient of friction (COF) and wear rate (δ) [38]. Jia et al. also verified through experiments that when N-P compound additives were used, N/P not only played a role in dispersion but also enriched more lubricating phosphates at the interface and formed a friction film with lower shear force and friction interface protection [39]. Synergistic mechanisms of composite additives are relatively complex and involve multiple factors such as membrane structures that prevent physical adsorption and frictional chemical reactions [39,40]. Thus, further investigation is warranted to describe their composite mechanisms of action.

In this study, the tribological properties of water-soluble molybdenum- and phosphorus-based additives were systematically investigated in EG aqueous solutions. A comparative analysis of additive concentrations revealed that the composite formulation of 0.5 wt.% Mo-based and 2.0 wt.% P-based additives yielded optimal performance, enhancing the lubricating efficiency and anti-wear capability by over 31% and 71%, respectively, while improving extreme-pressure resistance by nearly sixfold. These improvements substantially broaden the applicability of EG-based fluids under extreme operating conditions. Unlike single-additive systems, which offer limited functional enhancement, the present work elucidates the in situ formation of a structurally graded bilayer tribofilm—composed of a thin P_x_O_y_-rich inner layer and a thick amorphous carbon outer layer—as the key mechanism underlying the observed synergistic effect. By correlating tribofilm characteristics with macroscopic performance, this study provides a mechanistic foundation for the design of high-performance water–EG HFCs suitable for severe operational environments.

## 2. Materials and Methods

### 2.1. Experimental Materials

Dow EG solution (with a purity of over 99.9%) was purchased from Shanghai Kangmiao Trading Co., Ltd. (Shanghai, China) [11]. Water-soluble Mo (TM-104) was purchased from Chengsida Lubricant Co., Ltd. (Beijing, China) in the Liaocheng Development Zone, and the P-additive (VANLUBE 672) was purchased from Vanderbilt Chemical Co., Ltd. (Norwalk, CT, USA). GCr15 balls (diameter: 12.7 mm, surface roughness (S_a_) not exceeding 10 nm, hardness: 64–66 HRC) were purchased from Shanghai Steel Ball Co., Ltd. (Shanghai, China) [41]. The physical and chemical properties of the additives and the elemental composition and content of the GCr15 balls are listed in Table 1 and Table 2, respectively. Organic solvents (anhydrous ethanol, acetone, and petroleum ether) that met international standards and had purities greater than 99% were purchased from Shanghai Aladdin Biochemical Technology Co., Ltd. (Shanghai, China) [42]. Notably, the conductivity of ultrapure water is 18.2 MΩ·cm, and the total organic carbon content has been measured as less than 2 ppb [43].

### 2.2. Experimental Methods

Before the friction experiment, the GCr15 ball and oil cup fittings were ultrasonically cleaned with petroleum ether, acetone, and anhydrous ethanol for 15 min to remove any residual impurities from the surface. An MS-10A four-ball friction testing machine (Xiamen Tianji Automation Co., Ltd., Xiamen, China) was used to conduct tests on the long wear, variable load, variable speed, and extreme pressure performance. The optimal experimental conditions to select concentrations were 100 N (corresponding to a maximum Hertz contact pressure of 1440 MPa), 1200 rpm (corresponding to a linear velocity of 461 mm/s), and running at 25 °C for 30 min. The comparative experimental time between the different samples was extended to 60 min. The comparative samples were run for 10 min under the above conditions, and further variable load and speed experiments were conducted under loads of 50–200 N and speeds of 200–2400 rpm. According to the GB/T 12583 standard [44], the last non-seizure load (P_B_) and maximum sintering load (P_D_) of the comparative samples were measured separately [4]. All friction and wear tests were repeated at least three times to ensure the reliability of the experimental results. After the friction experiment, the GCr15 ball and oil cup fittings were cleaned with organic solvents for 5 min to remove surface lubricants and additive residues and then used for surface microstructure and composition characterization.

A three-dimensional (3D) white light morphology interferometer (Nexview; Zygo Lamda, Middlefield, CT, USA) was used to measure the surface morphology parameters of the worn area. The GCr15 ball *δ* was calculated using the following formula (1) [45].

(1) $$\delta \;=\frac{V}{F\cdot v\cdot T}$$

where *V* is the wear volume and *F*, *v*, and *T* are the load, linear speed, and friction processing time, respectively.

The microstructure and elemental distribution of the worn areas on the surfaces of the GCr15 balls were determined using a combination of cold-field emission scanning electron microscopy (FESEM) (SU8220, Hitachi, Tokyo, Japan) and energy-dispersive spectroscopy (EDS) (QUANTAX, Bruker, Berlin, Germany). The chemical composition and mechanical properties of the worn areas were analyzed using X-ray photoelectron spectroscopy (XPS) (PHI Quantera II, Ulvac-Phi, Chigasaki, Japan) and a nanoindentation instrument (TriboIndenter, Billerica, MA, USA). Transmission samples were analyzed with a focused ion beam scanning electron microscope (FIB-SEM) (LYRA3; TESCAN. Q.S., Brno, Czech Republic), and a high-resolution transmission electron microscope (HRTEM) (2100F, JEM, Akishima, Japan) was used to measure the thickness and composition of the friction reaction film on the cross section of the area [46].

## 3. Results and Discussion

### 3.1. Results

**Figure 1:**
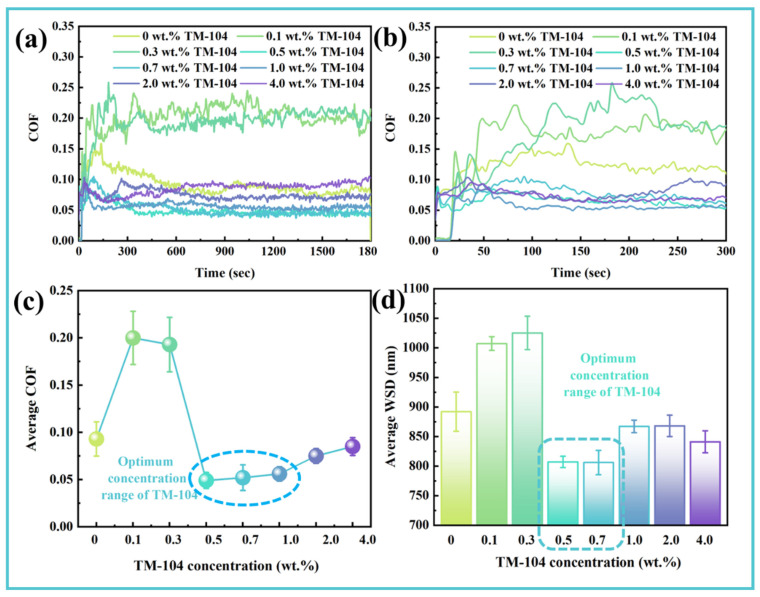
Tribological performance of ethylene glycol aqueous solutions with varying TM-104 concentrations: (**a**) Temporal evolution of the friction coefficient; (**b**) Enlarged view of the running-in period (0–300 s); (**c**) Comparison of the average friction coefficients; (**d**) Comparison of wear scar diameters.

A series of long-term friction tests were performed on ethylene glycol (EG) aqueous solutions (50 wt.% water content) incorporating water-soluble molybdenum-based additive TM-104 at varying concentrations. The temporal evolution of the COF is presented in Figure 1, along with the corresponding average COF and wear scar diameter (WSD) values. As shown in Figure 1a,b, at TM-104 concentrations below 0.3 wt.%, the COF exceeded that of the base EG solution and exhibited pronounced fluctuations over time. In contrast, at concentrations equal to or above 0.5 wt.%, the running-in period was substantially shortened, and the COF stabilized at values significantly lower than those of the additive-free solution. Figure 1c,d demonstrate that at TM-104 concentrations between 0.5 and 1.0 wt.%, the average COF was reduced by more than 40%. The antiwear performance, evaluated via WSD measurements, reached an optimum in the 0.5–0.7 wt.% range. Further increasing the additive concentration beyond 0.7 wt.% led to enlarged wear scars and accelerated wear. Based on systematic evaluation of the tribological behavior of EG solutions with different TM-104 loadings, the optimal concentration of this water-soluble molybdenum additive was determined to be 0.5 wt.%.

**Figure 2:**
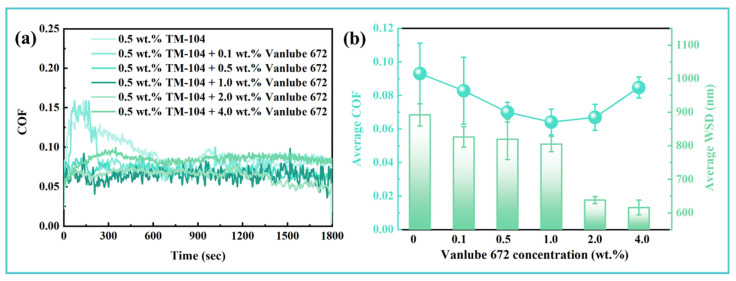
Synergistic effect of Vanlube 672 concentration on the tribological performance of ethylene glycol aqueous solution containing 0.5 wt.% TM-104: (**a**) Evolution of friction coefficient with time under continuous sliding; (**b**) Quantitative comparison of the average friction coefficient and wear scar diameter across different additive loadings.

While the addition of TM-104 alone was found to enhance the lubricating and anti-wear properties of the EG solution under mild conditions (100 N, 1200 rpm), its most pronounced effect was observed in the reduction in friction (Figure 1c,d). To further improve the tribological performance under more severe operating regimes, a synergistic approach combining water-soluble molybdenum-based TM-104 with phosphorus-containing Vanlube 672 was investigated. Long-term friction tests were conducted on EG solutions containing 0.5 wt.% TM-104 with varying concentrations of Vanlube 672, as summarized in Figure 2. The introduction of Vanlube 672 resulted in a stable initial friction coefficient without a pronounced running-in period (Figure 2a). The low amplitude fluctuation of the friction curve over time suggests the rapid formation of a durable adsorption film within the contact zone. Analysis of the average COF and WSD revealed that the optimal concentration of Vanlube 672 was 2.0 wt.% (Figure 2b). At this loading, both friction reduction and anti-wear performance reached their peak effectiveness. Further increases in additive content marginally improved anti-wear behavior but considerably compromised lubricity. Thus, the formulation with 0.5 wt.% TM-104 and 2.0 wt.% Vanlube 672 was identified as the optimal composite additive system for EG-based hydraulic fluids.

**Figure 3:**
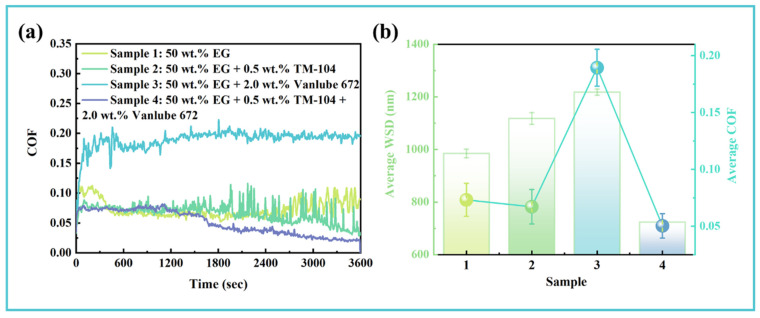
Long-term tribological performance of different lubricant formulations: (**a**) Temporal evolution of friction coefficients over 60 min testing duration; (**b**) Comparative analysis of average friction coefficients and corresponding wear scar diameters across all samples.

To evaluate the long-term tribological performance of the composite additive, comparative friction tests were conducted over 60 min under sustained grinding conditions. As illustrated in Figure 3a, the four samples exhibited distinct friction evolution behaviors. Sample 1 (baseline 50 wt.% EG solution) underwent a noticeable running-in period before reaching a temporarily stable friction state. However, after 2700 s, severe wear led to a sharp increase in the COF, attributable to the accumulation of wear debris in the contact zone [43]. Sample 2 (EG + 0.5 wt.% TM-104) showed a shortened running-in phase, but as the test progressed, the COF curve displayed frequent random fluctuations. These “protrusions” are associated with third-body abrasion caused by entrained wear debris [47]. After 30 min, the COF decreased to a relatively low level, suggesting the gradual formation of a friction-modifying surface film. Sample 3 (EG + 2.0 wt.% Vanlube 672) demonstrated inferior lubrication performance, exhibiting a high initial COF that stabilized after running-in, yet remained significantly above the baseline EG solution. In contrast, Sample 4 (EG + 0.5 wt.% TM-104 + 2.0 wt.% Vanlube 672) exhibited minimal running-in behavior and maintained the lowest and most stable COF throughout the test. A further reduction in COF after 1650 s indicates progressive formation and thickening of a protective tribofilm [48]. As summarized in Figure 3b, the WSD of single-additive formulations exceeded that of the base fluid after prolonged testing. However, the composite additive system reduced the average COF and WSD by 31.27% and 26.50%, respectively, confirming the synergistic enhancement of long-term tribological performance through additive combination.

**Figure 4:**
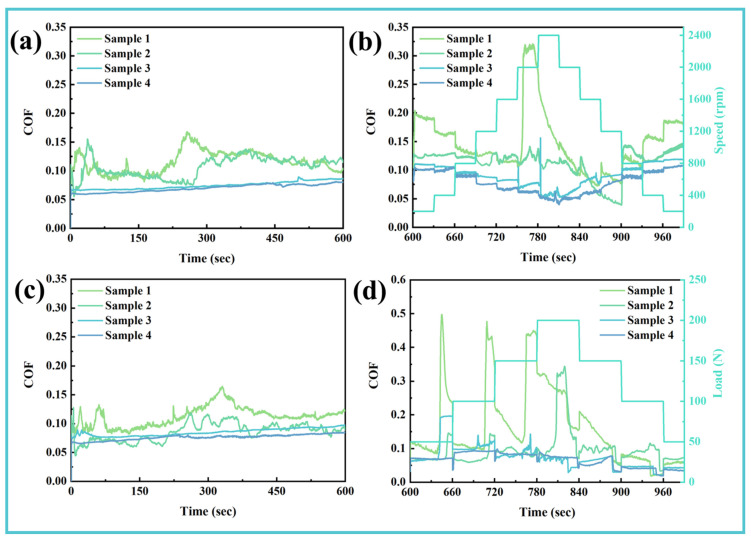
Lubricant adaptability to variable operating conditions: (**a**,**c**) Running-in behavior under baseline conditions (100 N, 1200 rpm, 10 min); (**b**,**d**) Dynamic response of friction coefficient to systematic variations in (**b**) rotational speed and (**d**) applied load following run-in completion.

To ensure reliable performance across diverse operational scenarios, lubricating media are required to maintain stable functionality under varying working conditions. In this study, the adaptability of the composite additive was evaluated through speed- and load-varying tests conducted after an initial run-in period. As shown in Figure 4a,c, the run-in behavior under baseline conditions (100 N, 1200 rpm, 25 °C) is consistent with the long-term friction results presented in Figure 3a. Following run-in, systematic variations in rotational speed (200–2400 rpm) and load (50–200 N) were introduced to examine the dynamic tribological response. As illustrated in Figure 4b,d, the COF of the additive-free Sample 1 exhibited pronounced instability, particularly under high-speed and high-load conditions, where repeated run-in behavior was observed before reaching temporary stabilization. Samples 2 and 3, formulated with single additives, showed improved but limited adaptability, maintaining stable COF only within narrow ranges of speed and load. In contrast, Sample 4, containing the composite additive system (0.5 wt.% TM-104 + 2.0 wt.% Vanlube 672), demonstrated consistently lower and more stable friction across the entire spectrum of tested conditions. These results confirm that the synergistic interaction between TM-104 and Vanlube 672 effectively expands the operational window and enhances lubrication stability under variable and severe service conditions.

**Figure 5:**
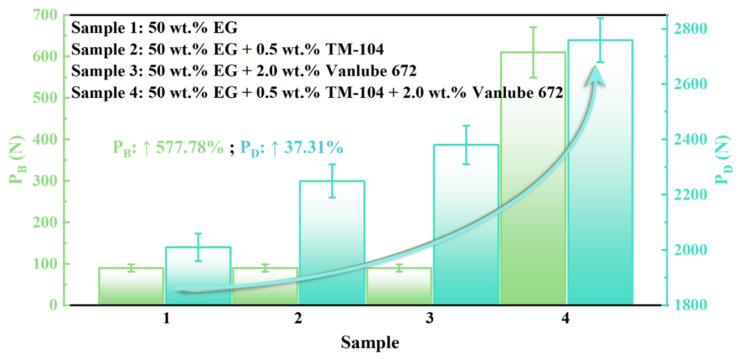
Extreme pressure performance evaluation: Comparison of the maximum non-seizure load (P_B_) and weld load (P_D_) values across different lubricant formulations.

The extreme pressure performance of different lubricant formulations was evaluated through measurement of the seizure load (P_B_) and weld load (P_D_), with results summarized in Figure 5. While single-additive formulations (Samples 2 and 3) showed no significant improvement in P_B_ value over the base EG fluid (Sample 1), the composite additive system (Sample 4) exhibited a remarkable enhancement—increasing the P_B_ value from 90 N to 610 N, representing a nearly sixfold (>577%) improvement. In terms of anti-weld performance, all additive-containing formulations increased the P_D_ value to varying degrees, with Sample 4 achieving the most substantial gain of 37.31% relative to the base fluid. These findings demonstrate that the synergistic combination of TM-104 and Vanlube 672 significantly enhances the load-carrying capacity and extreme pressure resistance of EG-based fluids, substantially expanding their operational limits in severe service environments and supporting more reliable application of HFC hydraulic fluids under extreme conditions.

### 3.2. Discussion

**Figure 6:**
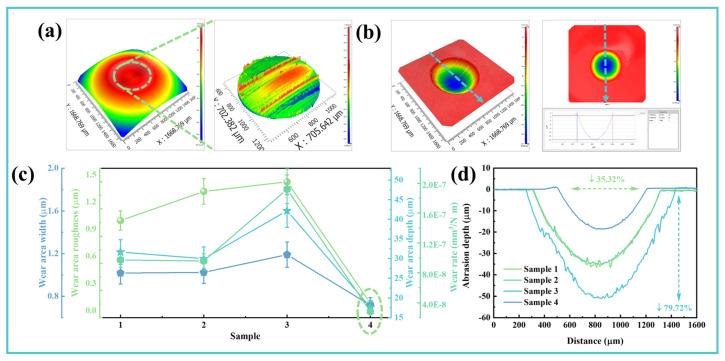
Multi-scale wear characterization of GCr15 balls after tribological testing: (**a**) Representative 3D white-light interferometry topography with detailed wear scar visualization (Sample 3); (**b**) Flattened surface morphology with schematic illustration of depth measurement methodology; (**c**) Quantitative comparison of surface roughness (S_a_), wear scar dimensions, and specific wear rate; (**d**) Cross-sectional depth profiles of wear tracks across all formulations.

To quantitatively evaluate the influence of additives on the wear behavior of ethylene glycol (EG) solutions, surface characterization of worn GCr15 steel balls was performed using white light interferometry. Key topographic parameters—including surface roughness (S_a_) within the wear scar, scar width, maximum depth, and wear volume—were systematically analyzed, with detailed results presented in Figure 6 and Table 3 [4,43]. The interferometric 3D topography of each spherical cap was acquired and subsequently flattened through digital processing to isolate wear-induced surface features, as illustrated in Figure 6a,b [49]. From these reconstructed surfaces, the scar width and Sa were directly extracted, while depth profiles and cross-sectional curves were derived to quantify wear penetration (Figure 6c,d). Analysis reveals that single-additive formulations not only failed to improve surface integrity but, in the case of Vanlube 672, even exacerbated surface damage. In marked contrast, Sample 4—containing the composite additive system—exhibited the optimal wear surface morphology, characterized by the smoothest topography and a substantial reduction in S_a_ from 1.072 μm to 0.159 μm. Relative to the baseline EG solution (Sample 1), the composite formulation reduced scar width and maximum depth by 35.32% and 79.72%, respectively; decreased wear volume by over 71%; and achieved a specific *δ* of 2.81 × 10^−8^ mm^3^/N·m, as indicated by the blue dashed line in Figure 6c.

**Figure 7:**
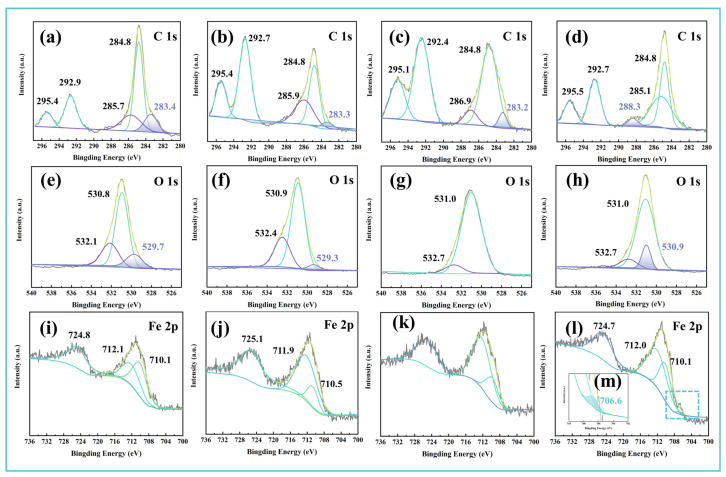
Chemical state analysis of wear tracks via X-ray photoelectron spectroscopy: High-resolution spectra of (**a**–**d**) C 1s, (**e**–**h**) O 1s, and (**i**–**l**) Fe 2p regions for Samples 1–4, respectively. (**m**) is a partial enlargement of (**l**).

XPS was employed to characterize the chemical states of key elements (C, O, Fe) within the wear tracks of different samples, as shown in Figure 7. In the C 1s spectra, characteristic peaks were observed at 284.8 eV (C–C), 285.7 eV (C–O), and 288.4 eV (C=O), confirming the presence of adsorbed lubricant-derived films in all wear zones [50,51]. Notably, in Sample 4 (Figure 7a–d), the C–C peak at 283.2 eV disappeared, while the C=O peak intensity increased significantly, indicating substantial tribo-chemical oxidation during friction. In the O 1s spectra (Figure 7e,f), Samples 1 and 2 exhibited a dominant peak at 529.7 eV, consistent with iron oxide formation [23]. With the addition of Vanlube 672 (Sample 3), a phosphate-related peak (PO_4_^3−^) emerged at 532.7 eV, which was replaced in Sample 4 by a new oxide peak at 530.9 eV, suggesting a shift in reaction products (Figure 7g,h). Corresponding Fe 2p spectra revealed a distinct peak at 706.6 eV exclusively in Sample 4, attributable to iron phosphide or similar phosphorous-containing iron species [52] (Figure 7i,m).

**Figure 8:**
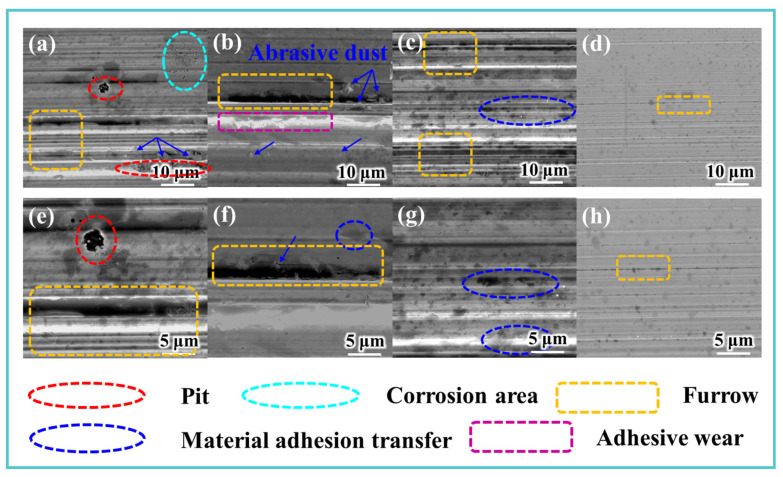
Surface morphology of wear tracks: (**a**–**d**) SEM images, (**e**–**h**) high-magnification SEM details.

These spectral changes collectively demonstrate that the TM-104 + Vanlube 672 composite additive promotes the in situ formation of a complex tribofilm comprising oxidized carbon species, iron oxides, and phosphorus-containing compounds. Surface morphology and elemental composition of the wear tracks were characterized using SEM coupled with energy-dispersive X-ray spectroscopy (EDS), as summarized in Figure 8. The wear surface of Sample 1 (Figure 8a,e) exhibited pronounced plowing grooves accompanied by extensive material delamination and corrosion pits, indicating dominant abrasive and corrosive wear mechanisms. In Samples 2 and 3, formulated with single additives, the persistence of deep grooves was observed alongside substantial adhesive material transfer, reflecting a combination of adhesive and abrasive wear mechanisms (Figure 8b,c,f,g). In contrast, Sample 4—containing the composite additive system—displayed a notably smoother wear surface with shallower grooves and an absence of significant delamination or adhesive transfer (Figure 8d,h). The predominant wear mechanism in this case was mild abrasive wear, demonstrating the superior protective effect of the synergistic additive formulation.

**Figure 9:**
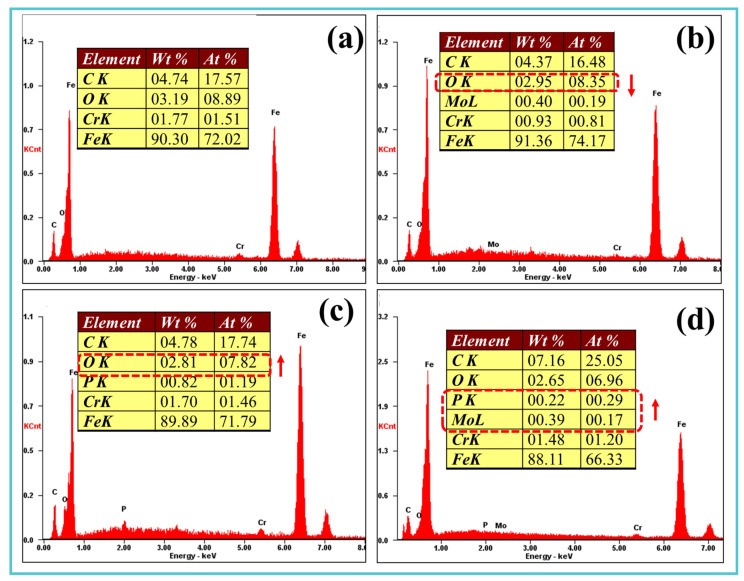
Elemental composition of wear tracks: (**a**–**d**) EDS elemental mappings of Samples 1–4 in Figure 8, respectively.

**Figure 10:**
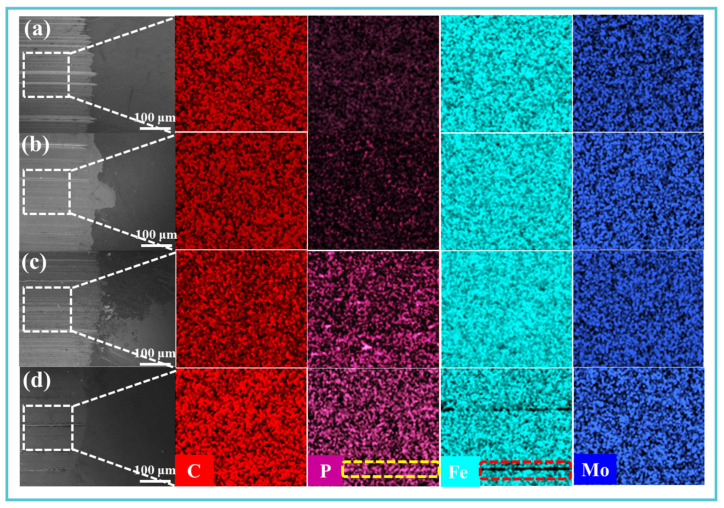
Localized wear scar characterization: SEM morphology and corresponding elemental distribution mappings of representative wear areas for (**a**) Sample 1, (**b**) Sample 2, (**c**) Sample 3, and (**d**) Sample 4.

EDS analysis of the worn surfaces revealed substantial enrichment of Mo, P, and C in Samples 2–4, confirming effective additive-derived tribofilm formation (Figure 9a,b). In Sample 4 (Figure 9d), the concentrations of Mo and C reached 0.39 wt.% and 7.16 wt.%, respectively, while P content (0.22 wt.%) remained significant despite being lower than in Sample 3. Elemental mapping (Figure 10) further demonstrated pronounced P segregation within the plowing grooves of Sample 4, accompanied by a corresponding decrease in Fe signal. This redistribution of elements confirms the synergistic effect of TM-104 and Vanlube 672 in generating a protective tribofilm, which is critical for the superior antiwear performance observed in Sample 4.

**Figure 11:**
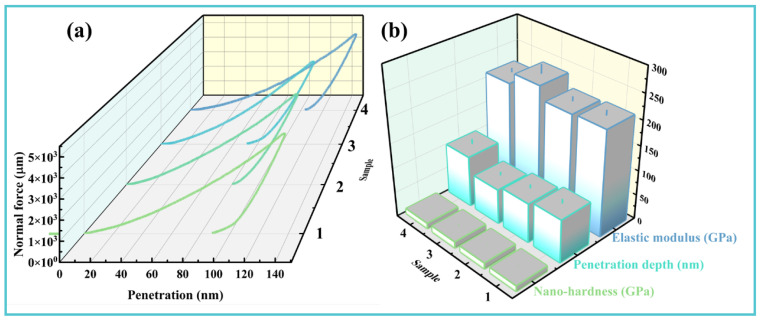
Nanomechanical properties of wear tracks: (**a**) Nanoindentation load–displacement curves and (**b**) comparative analysis of nanohardness, residual indentation depth, and elastic modulus across different lubricant formulations.

To elucidate the role of tribofilm mechanical properties in antiwear performance, nanoindentation measurements were performed within the wear tracks of different samples (Figure 11). As shown in Figure 11a, under a 5 mN indentation load, Samples 1–3 exhibited relatively shallow penetration depths (~130 nm), indicating higher surface hardness. In contrast, Sample 4 displayed a greater indentation depth of approximately 150 nm, suggesting the presence of a softer surface layer formed by tribochemical reactions [46]. Quantitative analysis of the mechanical properties (Figure 11b) revealed that the tribofilm formed in Sample 4 reduced both nanohardness and elastic modulus to 10.75 GPa and 205.94 GPa, respectively, compared to 11.72 GPa and 214.07 GPa for the additive-free Sample 1. This mechanical property modification, coupled with the previously established presence of P- and C-rich tribofilms (Figure 9d), indicates that the composite additive promotes the formation of a shear-compliant surface layer. This engineered interface effectively prevents direct asperity contact between friction pairs, reduces shear resistance, and thereby enhances overall tribological performance—including lubricity, wear resistance, and extreme pressure capacity.

**Figure 12:**
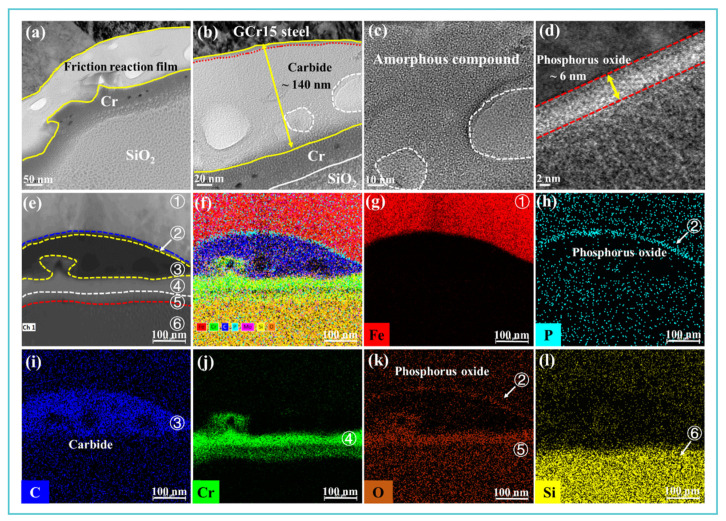
Microstructure and elemental composition of the synergistic tribofilm generated by the composite additive (Sample 4): (**a**–**d**) Cross-sectional TEM visualization of the ~140 nm amorphous carbon/~6 nm P-rich oxide bilayer; (**e**–**l**) EDS mapping confirming the stratified distribution of key elements, elucidating the film’s self-organized structure.

Multimodal characterization provided conclusive evidence for the formation and structure of a synergistic tribofilm. SEM-EDS and nanoindentation confirmed a P-/Mo-/C-containing film with reduced shear strength (Figure 8, Figure 9 and Figure 11). Cross-sectional HRTEM of the wear track (Sample 4, Figure 12) further resolved a hierarchically structured tribofilm, comprising a thick (~140 nm) amorphous carbon top layer and an ultrathin (~6 nm) P-rich oxide layer at the substrate interface (Figure 12a–d). The observed ion beam damage within the amorphous carbon region (white dashed boxes, Figure 12b,c) underscores the structural distinction between the two sublayers.

High-resolution TEM and elemental mapping analysis (Figure 12e–l) provided a stratified characterization of the tribofilm formed by the composite additive (Sample 4) on the GCr15 ball surface. The cross-section was divided into six distinct regions, each exhibiting specific structural and compositional features: Region ① corresponds to the GCr15 substrate, dominated by Fe (Figure 12g), which serves as the structural foundation of the friction pair. Region ② consists of a nanoscale P_x_O_y_ compound layer with significant enrichment of P and O elements (Figure 12h,k). This layer, although only approximately 6 nm thick, is uniform and dense, and acts as a critical barrier against direct asperity contact in the friction zone, contributing substantially to the superior antiwear performance [46,53]. Region ③ comprises a relatively thick (~140 nm) amorphous carbon film (Figure 12i), which effectively reduces shear stress in the contact area and enhances both lubricity and wear resistance. The improved tribological performance under extreme pressure is attributed to mechanisms such as atomic-level structural ordering, formation of thin-layer shear bands, and shear-induced covalent bond alignment along the sliding direction [54]. Regions ④ and ⑥ represent the Cr and SiO_2_ protective layers, respectively, while Region ⑤ denotes the transition layer between them (Figure 12j–l). It is worth noting that the molybdenum content in regions ② and ③ is still relatively low, which indicates that the Mo element did not directly participate in the formation of the friction reaction film. The results clearly show that when TM-104 is present, nano-layer P_x_O_y_ and amorphous carbon films are effectively generated on the surface.

**Figure 13:**
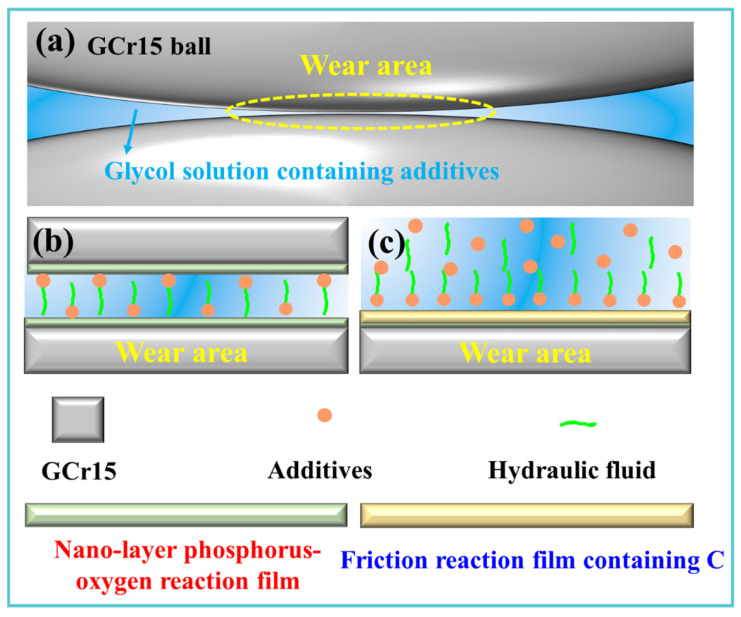
Mechanistic model of tribofilm formation and lubrication. The diagram contrasts (**a**,**b**) the ineffective adsorption with single additives against the synergistic in situ generation of a sandwich architecture—comprising (**c**) a thin P_x_O_y_ layer and a thick amorphous carbon film—which collectively enhances lubricity and load-bearing capacity under boundary lubrication.

Based on the experimental results, the antifriction and antiwear mechanisms of the composite additive in the EG solution are proposed in Figure 13. In the presence of additives, the surface of the GCr15 ball experiences measurable wear in the contact area (Figure 13a). When only Vanlube 672 is present, the additive competes with EG–H_2_O molecules for adsorption sites at the friction interface, forming an alternating physical adsorption film. This film disrupts the stable EG–H_2_O hydrogen-bonding network, weakening interfacial interactions and leading to the relatively high average COF and WSD observed in Figure 3. Under severe wear conditions, a discontinuous phosphorus-containing tribofilm forms locally (Figure 13b) [4]. However, the high hardness and strong shear effects in the wear zone result in significant abrasive wear. When both TM-104 and Vanlube 672 are present, their synergistic interaction markedly enhances the tribological performance of the EG solution (Figure 3). Initially, MoO_4_^2−^ anions from TM-104 adsorb onto the positively charged metal surface, forming an anionic layer, while Mo-containing cations arrange into an ordered multilayer adsorption structure [40]. The alkyl chains of the phosphorus-based additive and the nonpolar end groups of the molybdenum compound further enhance this process, leading to a thicker physical adsorption layer. TM-104 promotes the stabilization of the adsorption film from Vanlube 672, and under shear, a tribochemical reaction rapidly generates a nanoscale P_x_O_y_ film. Nitrogen species from TM-104 likely play a key role by decomposing into organic amines that adsorb on nascent metal surfaces and act as dispersants [37,55]. Concurrently, phosphate additives contribute to forming a dense, wear-resistant reaction film. The in situ formation of the bilayer tribofilm is attributed to the extreme conditions—high temperature, pressure, and shear—in the contact zone, which trigger chemical reactions at freshly exposed active sites [39]. The resulting film is dense and effectively covers the substrate, preventing direct asperity contact. With continued friction, a thicker (~140 nm) amorphous carbon layer forms above the P_x_O_y_ interlayer (Figure 13c). This structure reduces nanohardness and elastic modulus, mitigates shear stress, promotes interfacial covalent bond alignment, and facilitates shear band formation. In summary, the composite additive system leads to the in situ formation of a “functionally graded structure” in the contact area, comprising a thin, hard P_x_O_y_ underlayer and a thick, soft amorphous carbon top layer, separated by a lubricating medium. This architecture effectively prevents direct contact between friction pairs, significantly improving the lubrication, antiwear, and extreme-pressure properties of the EG solution under boundary lubrication conditions.

**Figure 14:**
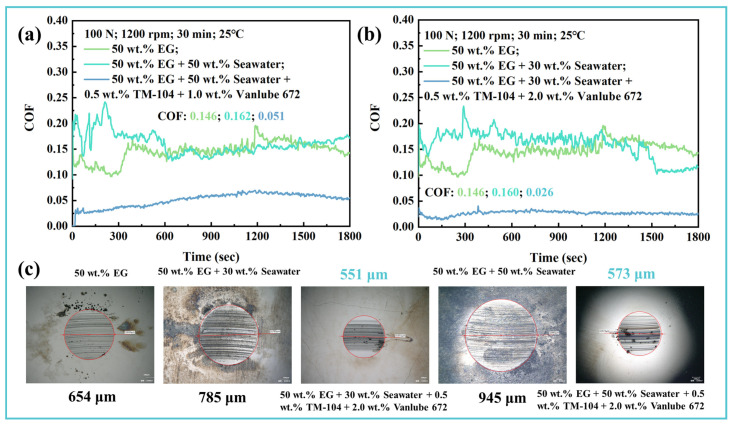
Corrosion-resistant tribological performance of the composite additive system. (**a**,**b**) Friction stability over time in 30 wt.% and 50 wt.% seawater, respectively; (**c**) Representative wear scar image, confirming the integrity of the protective tribofilm despite ionic competition and viscosity reduction. Magnification ×200.

EG solution, serving as the primary component of HFCs, is extensively employed in marine hydraulic systems. However, the inevitable infiltration of seawater into these systems significantly compromises the tribological performance of HFCs [4]. Seawater ingress reduces the viscosity of the lubricating medium, promoting a transition to boundary lubrication conditions. Furthermore, hydrated ions from seawater compete with lubricant molecules for adsorption sites, disrupting the EG–H_2_O physical adsorption film and accelerating wear. In this study, we evaluated the efficacy of the 0.5 wt.% TM-104 + 2.0 wt.% Vanlube 672 composite additive under simulated seawater conditions (Figure 14). The results demonstrate a substantial improvement in lubricating performance, with the COF reduced to 0.026 and 0.051 at 30 wt.% and 50 wt.% seawater concentrations, respectively (Figure 14a,b). Moreover, the WSD decreased by over 30% from 785 μm and 945 μm to 551 μm and 573 μm under the same conditions (Figure 14c). These findings confirm that the composite additive effectively enhances the lubricity, wear resistance, and extreme-pressure performance of EG-based fluids, even in seawater environments, thereby supporting the reliable application of HFCs in marine equipment. Although dissolved ions can reduce the viscosity of the lubricating medium, the adsorption of hydrated ions will increase the physical adsorption layer on the surface, thereby reducing the shear effect and effectively lowering COF [43]. This study provides new insights into the influence of salt ions on tribofilm formation and establishes a practical basis for screening high-performance composite additives for seawater-compatible hydraulic fluids. These findings significantly broaden the potential applications of HFCs in challenging marine environments.

## 4. Conclusions

This study establishes that the synergistic combination of TM-104 and Vanlube 672 significantly enhances the tribological performance of EG aqueous solutions through the in situ formation of a nanoscale bilayer tribofilm. The key findings are summarized as follows:

(1) The optimized formulation with 0.5 wt.% TM-104 and 2.0 wt.% Vanlube 672 reduced the friction coefficient and specific wear rate by over 31% and 71%, respectively, while increasing the P_B_ and P_D_ by 577% and 37%.
(2) Cross-sectional characterization confirmed the presence of a structurally graded tribofilm, consisting of a ~6 nm thick P_x_O_y_-rich inner layer and a ~140 nm thick amorphous carbon outer layer. This architecture effectively reduces shear resistance and prevents direct asperity contact.
(3) The first verification showed that when TM-104 and Vanlube 672 additives were combined, they could promote the formation of a “functionally graded structure” through a synergistic adsorption agent friction chemical reaction. This structure mainly consists of a ~6 nm P_x_O_y_ film and a ~40 nm amorphous carbon film.

These results provide a mechanistic foundation for designing high-performance fire-resistant hydraulic fluids capable of operating under extreme conditions, while demonstrating the potential of molecularly engineered additive systems to enable stable lubrication in challenging environments such as seawater-contaminated systems.

## Figures and Tables

**Table 1:** Suppliers of additives and their physicochemical parameters.

Additive; Supplier	Density (kg/m^3^)	Kinematic Viscosity (100 °C, mm^2^/s)	Flash Point (°C)
Vanlube 672; Vanderbilt Chemicals, LLC (Beijing, China)	1.02	250	113
TM-104; Chengstar Lubricant Co., Ltd. (Shangdong, China)	0.95	500	355

**Table 2:** GCr15 steel elemental compositions.

GCr15 Steel Elemental Compositions					
Compositions	Fe	C	Mn	Si	S
Content	Balance	0.95–1.05	0.25–0.45	0.15–0.35	≤0.025
Compositions	P	Cr	Mo	Ni	Cu
Content	≤0.025	1.40–1.65	≤0.10	≤0.30	≤0.25

**Table 3:** Quantified wear parameters of GCr15 balls post-tribological testing: WSD, S_a_, wear scar width, wear scar depth, wear volume, and specific *δ*.

GCr15 Balls	Sample 1	Sample 2	Sample 3	Sample 4
WSD (µm)	985	1118	1218	724
S_a_ (µm)	1.072	1.394	1.501	0.159
Wear area width (µm)	1.017	1.023	1.190	0.716
Wear area depth (µm)	31.673	29.979	42.181	17.624
Wear volume (µm^3^)	1.32 × 10^7^	1.30 × 10^7^	2.60 × 10^7^	3.80 × 10^6^
*δ* (mm^3^/N·m)	9.72 × 10^−8^	9.58 × 10^−8^	1.92 × 10^−7^	2.81 × 10^−8^

## Data Availability

The original contributions presented in this study are included in the article. Further inquiries can be directed to the corresponding authors.

## Abbreviations

Water-glycol fire-retardant hydraulic fluid (HFC); ethylene glycol (EG); surface roughness (S_a_); last non-seizure load (P_B_); maximum sintering load (P_D_); wear rate (*δ*); field emission scanning electron microscopy (FESEM); energy dispersive spectroscopy (EDS); X-ray photoelectron spectroscopy (XPS); focused ion beam scanning electron microscope (FIB-SEM); high-resolution transmission electron microscopy (HRTEM); coefficient of friction (COF); wear spot diameter (WSD).
